# Scorpion and spider venoms in cancer treatment: state of the art, challenges, and perspectives

**Published:** 2017-05-24

**Authors:** Catarina Rapôso

**Affiliations:** Department of Structural and Functional Biology, Institute of Biology, State University of Campinas (UNICAMP), Campinas, SP, Brazil

**Keywords:** Spider venom, scorpion venom, toxins, cancer therapy, cancer mechanism, translational research

## Abstract

**Background and Aim:** Animal venoms comprise a mix of bioactive molecules with high affinity for multiple targets in cells and tissues. Scorpion and spider venoms and purified peptides exhibit significant effects on cancer cells, encompassing four potential mechanisms: 1) induction of cell cycle arrest, growth inhibition, and apoptosis; 2) inhibition of angiogenesis; 3) inhibition of invasion and metastasis; and 4) blocking of specific transmembrane channels. Tumor biology is complex and entails many intertwined processes, as reflected in the putative hallmarks of cancer. This complexity, however, gives rise to numerous (potential) pharmacological intervention sites. Molecules that target multiple proteins or pathways, such as components of animal venoms, may therefore be effective anti-cancer agents. The objective of this review was to address the anti-cancer properties and in vitro mechanisms of scorpion and spider venoms and toxins, and highlight current obstacles in translating the preclinical research to a clinical setting.

**Relevance for patients:** Cancer is a considerable global contributor to disease-related death. Despite some advances being made, therapy remains palliative rather than curative for the majority of cancer indications. Consequently, more effective therapies need to be devised for poorly responding cancer types to optimize clinical cancer management. Scorpion and spider venoms may occupy a role in the development of improved anti-cancer modalities.

## 1 Introduction

Animal venoms are a mix of bioactive molecules that have a high affinity for multiple targets in prey or enemy organisms [1]. In spite of their toxicity, they can be used to investigate physiological and pathological processes and represent promising guiding compounds for drugs [2]. Single target interventions are largely ineffective in the treatment of complex systemic diseases, such as neurodegenerative diseases, AIDS, and cancer [3, 4]. In these cases, molecules that target numerous proteins or pathways involved in a disease, which include components of animal venoms, may be more effective than single-target therapies.

**Figure 1:**
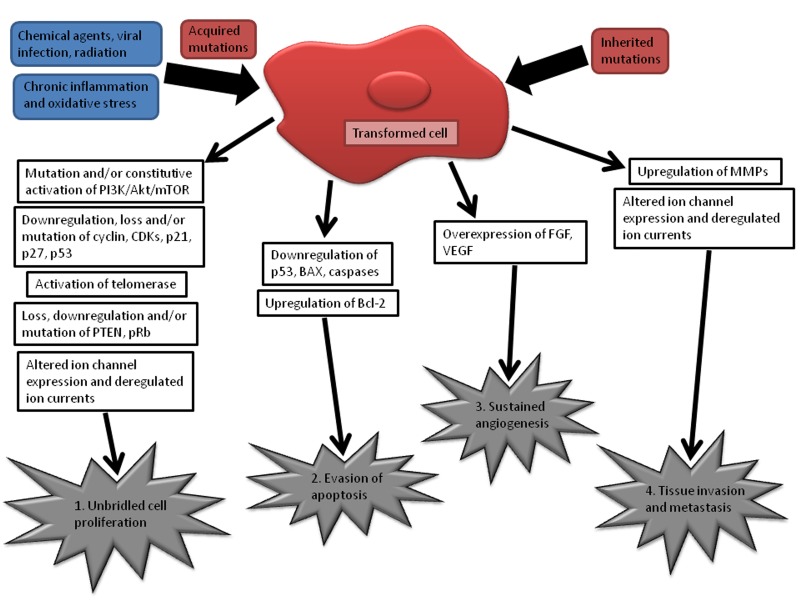
Schematic representation of the hallmarks of cancer development (1. deregulated cell proliferation; 2. evasion of programmed cell death; 3. sustained angiogenesis; 4. tissue invasion and metastasis) and the most important mechanisms accessed by scorpion and spider venoms on cancer cells. PI3K - phosphatidylinositol-3 kinase, Akt - protein kinase B, mTOR - mammalian target of rapamycin, CDKs – cyclin-dependent kinases, p21 and p27 - CDK inhibitors, PTEN - phosphatase and tensin homolog deleted on chromosome ten, pRb - Rb tumor-suppressor protein, Bcl-2 – B-cell lymphoma 2 (apoptosis regulator), FGF – fibroblast growth factors, VEGF – vascular endothelial growth, MMPs – matrix metalloproteinases.

**Figure 2:**
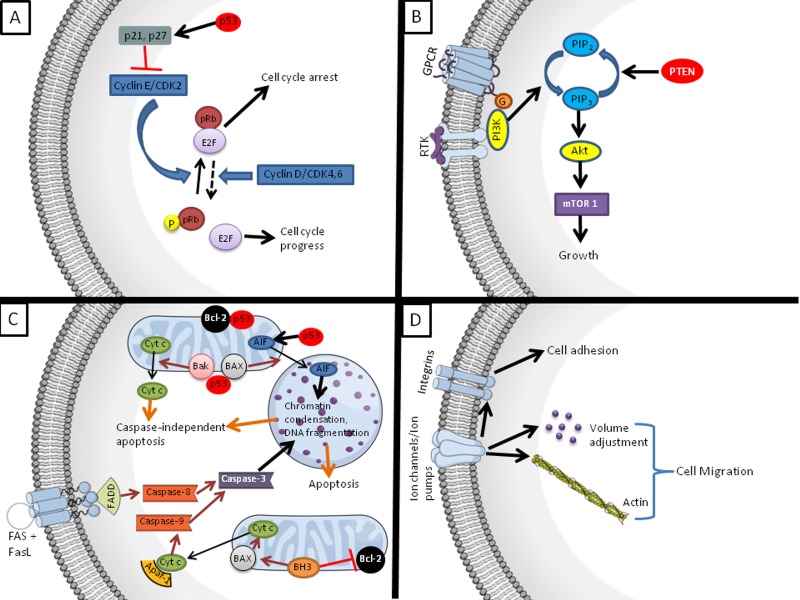
Schematic representation of the mechanisms involved in normal cell cycle control, growth, apoptosis, and cell migration/adhesion that are impaired in cancer development. The targets of these pathways are accessed by scorpion and spider venoms and toxins (described throughout the text). The pathways were presented in a simplified manner and several crosstalk and components were omitted. (A) The control of the cell cycle is regulated by the activity of cyclin dependent kinases (CDKs) and their essential activating coenzymes, the cyclins, and CDKs inhibitors (CDKIs). The phosphoprotein pRb (Rb tumor-suppressor protein) regulates the activity of the E2F transcription factor. Complexes consisting of E2F and hypophosphorylated pRb repress the transcription of the genes required for cell cycle progression. In contrast, phosphorylated pRb (by cyclin/CDK complexes) is unable to bind to E2F, resulting in the activation of E2F-dependent transcription and advancement into the late G1 and S phases. The p53-inducible proteins p21 and p27 (CDKIs) inactivate the cyclin/CDK complexes, leading to the dephosphorylation of pRb and cell cycle arrest. (B) Following activation by receptor tyrosine kinases (RTK) or G-protein-coupled receptors (GPCR), phosphatidylinositol-3 kinase (PI3K) catalyzes the phosphorylation of phosphatidylinositol 4,5-bisphosphate (PIP2) to generate phosphatidylinositol 3,4,5-trisphosphate (PIP3), which binds and recruits protein kinase B (Akt). Akt regulates cell growth by phosphorylation of the downstream mammalian target of rapamycin 1 (mTOR1), which promotes the translation of mRNAs to synthesize proteins. As a catalytic antagonist of PI3K, phosphatase and tensin homolog deleted on chromosome ten (PTEN) dephosphorylates PIP3 to PIP2. (C) At the top of the figure, the scheme represents the caspase-independent apoptosis mediated by p53. Activated p53 induces apoptosis by transactivating pro-apoptotic genes (e.g., BAX, Bak) and by also directly binding to anti-apoptotic mitochondrial proteins (e.g., Bcl-2). The p53 protein also activates apoptosis-inducing factor (AIF), a factor released from mitochondria to the nucleus, triggering large-scale DNA fragmentation and nuclear chromatin condensation. In the lower part of the figure, the extrinsic and intrinsic canonical caspase-mediated apoptosis are depicted. In the extrinsic pathway, the death receptor-ligand (represented by FAS-Fas ligand - FAS + FASL) binds to the Fas-associated protein with death domain (FADD), constructing a complex called the death-inducing signaling complex, which activates initiator pro-caspase-8. Caspase-8 activates caspase-3, inducing apoptosis. The intrinsic apoptotic pathway is characterized by mitochondrial change in response to various stress signals, such as severe genetic damage, hypoxia, and oxidative stress, which activate the initiator pro-caspase-9. Mitochondrial pro-apoptotic proteins, BH3-only members, activate other pro-apoptotic proteins, such as BAX, and antagonize anti-apoptotic proteins (Bcl-2). Subsequently, the mitochondrial outer membrane is disrupted, and its permeability increases, resulting in cytochrome-c (Cyt-c) leakage into the cytosol. Cyt-c in cytosol forms a complex with Apaf-1, called the apoptosome, which assists in auto-activation of initiator pro-caspase-9. Caspase-9 activates caspase-3, leading to apoptosis. (D) Ion channels (Na+, K+, Cl−, Ca+) and ion pumps (Na+/K+-ATPase) promote cell migration through their ability to cause volume changes and by interacting with F-actin. Also, channels and pumps interact with integrins, leading to cell adhesion and facilitating migration. See [16, 107, 108, 109, 110] for a comprehensive review.

The development of cancer involves four categorical hallmarks (Figure 1): 1) dysregulated cell proliferation (due to the self-sufficiency of growth signals or insensitivity to growth inhibitory signals); 2) evasion of programmed cell death; 3) sustained angiogenesis; and 4) tissue invasion and metastasis [5, 6]. These characteristics are a consequence of DNA mutations which can be inherited or acquired (caused by e.g., virus and substance exposure, chronic inflammation, and oxidative stress) [7]. These DNA mutations trigger complex signals, signaling pathways, and crosstalk between signaling cascades [6] that are responsible for carcinogenesis, cancer cell proliferation, and metastasis [7]. Several pertinent molecular mechanisms that are impaired in cancer cells are illustrated in Figure 2. Finding molecules that can interact with multiple target/pathways and act on several hallmarks of cancer is one of the main challenges in anti-cancer pharmacology.

Today, several natural agents or their synthetic analogues are clinically prescribed for the treatment of cancer [8]. Of 98 new anticancer drugs approved by the US Food and Drug Administration (FDA) between 1981 and 2010, 78 were natural products or were derived from natural products, and only 20 were synthetic [9]. Despite their potential for use in the treatment of cancer, animal-derived molecules (mainly arthropods) are rarely used as drug prototypes or in clinical trials and practice.

The main objective of this review was therefore to address the effects and in vitro mechanisms of multi-targeting animal venoms, namely scorpion and spider venoms or their isolated substances (toxins), in relation to cancer. Moreover, the difficulties with translating the use of these molecules to the clinical setting are discussed.

## 2 Effect of scorpion and spider venoms on cancer cells

Biomolecules in scorpion and spider venoms have been shown to affect the abovementioned hallmarks of cancer, as summarized in Table 1. A more detailed account of the anti-cancer mechanisms is provided in the following sections.

### 2.1 Scorpion venom

**Figure 3:**
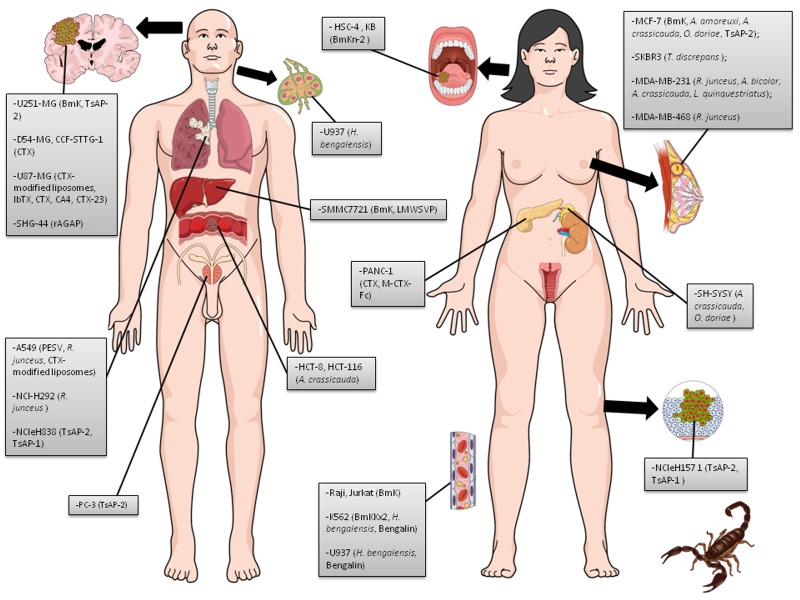
Human cancer cell lines used in scorpion venom and peptide studies in vitro. Images of both woman and man were inserted to represent cancers derived from the reproductive organs. There are no differences related to other lines in terms of gender. Each cell line is followed by the venom/peptide tested (in parentheses).

Scorpion venom is a complex mixture of protein (enzymes and peptides) and non-protein (inorganic salts, lipids, nucleotides, free amino acids, and water) substances produced by the venom gland for defense and capture of prey [10, 11]. An increasing number of experimental and preclinical investigations have demonstrated that crude scorpion venom and some purified proteins and peptides can impair multiple hallmarks of cancer (Figure 2) in vitro and in vivo. The effect and efficacy of scorpion venoms have been tested in glioma-, neuroblastoma-, leukemia-, lymphoma-, breast-, lung-, hepatoma-, pancreatic-, prostate-, and other models of cancer (Figure 3 and 5, Table 1). Only a few purified toxins seem to be responsible for the anticancer effects. These observations attest to the potential use of scorpion venoms and toxins in cancer therapy.

**Table 1:** Promising anticancer venom/toxins from scorpion and spider.

Specie	Compound	Target/Mechanism	Effect on cancer hallmarks*	*In vitro* cancer cell lines and *in vivo* tumor models•
**Scorpion**				
*Buthus martensii* Karsch (BmK)	Whole venom	Up-regulates caspase 3; Arrests cell cycle on G0/G1; Decreases Cyclin D1; Increases PTEN, p27	1, 2	Human glioma (U251-MG)•; Human lymphoma (Raji and Jurkat); Human breast cancer (MCF-7); Human hepatoma (SMMC7721)
	PESV	Decreases PI3K, Akt; Increases PTEN; Arrests cell cycle on G0/G1; Decreases mTOR; Reduces VEGF; Decreases microvessel density	1, 2, 3	Human leukemia (K562); Murine hepatoma (H2-2)•; Human lung (A549)
	BmKn-2	Increases caspase-3, 7, 9; decreases Bcl-2; Increases p53 and BAX	2	Human oral squamous carcinoma (HSC-4); Human mouth epidermoid carcinoma (KB)
	LMWSVP	Increases caspase-3; Decreases Bcl-2	2	Human hepatoma (SMMC7721)
	GST-BmKCT	Blocks Cl^-^ channel; Reduces MMP-2	1, 4	Rat glioma (C6)•
	Ad-BmKCT	Blocks Cl^-^ channel; Reduces MMP-2	1, 4	Rat glioma (C6)•
	rAGAP	Inhibit proliferation; Suppress migration; Arrest cell cycle on G1; Suppress CDK2, CDK6, pRb; Reduce pAkt, VEGF and MMP-9	1, 3, 4	Human anaplastic astrocytoma (SHG-44); Rat glioma (C6)
	BmKKx2	Blocks K^2+^ channels; Suppressed proliferation; Inhibits differentiation; Promotes differentiation-dependent apoptosis	1, 2	Human myelogenous leukemic (K562)
	TM-601	Blocks Cl^-^ channel	4	Rat glioma (F98); Human glioblastoma (U87)
*Androctonus amoreuxi*	Whole venom	Increases caspase-3; Induces DNA fragmentation; Reduces VEGF; Decreases cell motility and colony formation	2, 3, 4	Ehrlich ascites and solid tumors•; Human breast cancer (MCF-7)
*Androctonus crassicauda*	Whole venom	Increases caspase-3; Arrests cell cycle on S-phase; Depolarizes mitochondrial membrane; Decreases cell motility and colony formation	1, 2, 4	Human neuroblastoma (SH-SYSY); Human breast cancer (MCF-7); Human ileocecal adenocarcinoma (HCT-8); Human colorectal carcinoma (HCT-116); Human breast carcinoma (MDA-MB-231)
	Acra3	------	2	Mouse brain tumor (BC3H1)
*Heterometrus bengalensis Koch*	Whole venom	Arrests cell cycle; Induces membrane blabbing, chromatin condensation, DNA degradation	1, 2	Human leukemic (U937, K562)
	Bengalin	Induces DNA fragmentation; Decreases telomerase activity; loss of mitochondrial membrane potential; activates caspase-3, 9	2	Human leukemic (U937, K562)
*Tityus discrepans*	Whole venom, neopladine 1 and neopladine 2	Induce FasL expression and DNA fragmentation	2	Human breast (SKBR3)
*Odontobu-thus doriae*	Whole venom	Induces mitochondria depolarization and increases caspase-3	1, 2	Human neuroblastoma (SH-SYSY); Human breast (MCF-7)
*Rhopalurus junceus*	Whole venom	Induces chromatin condensation; Increases p53, caspases 3, 8, 9; decreases *bcl*-2 mRNA	2	Human lung (A549, NCI-H292); Human breast (MDA-MB-213, MDA-MB-468)
*Androctonus bicolor*	Whole venom	Decreases cell motility and colony formation	4	Human breast carcinoma (MDA-MB-231)
*Leiurus quinquestriatus*	Whole venom	Decreases cell motility and colony formation	4	Human breast carcinoma (MDA-MB-231)
	Chlorotoxin (CTX)	Inhibits/reduces MMP-2, inhibits Cl- currents	4	Human glioma (D54-MG, CCF-STTG-1); Human pancreatic carcinoma (PANC-1)
	GST-CTX	Inhibits/reduces MMP-2, inhibits Cl- currents	1, 4	Rat glioma (C6)•
	M-CTX-Fc	Inhibits/reduces MMP-2, inhibits Cl- currents	4	Human pancreatic carcinoma (PANC-1)
	CTX-modified liposomes	Inhibits/reduces MMP-2; Inhibits cell migration; Inhibits Cl^-^ currents	4	Human glioblastoma (U87); Human lung carcinoma (A549); Murine breast (4T1)•
	CA4 and CTX-23	Inhibit growth, membrane extensions and filopodia motility and migration; Inhibit angiogenesis	1, 4	Rat glioma (F98); Human glioblastoma (U87)
*Tityus serrulatus*	TiTx gamma	Affects Na^+^ channels	------	Mouse neuroblastoma (NIE115)
	TsIV-5	Blocks Na^+^ current	------	Mouse neuroblastoma (N18)
	TsAP-2	------	1	Human squamous carcinoma (NCIeH157); Human lung adenocarcinoma (NCIeH838); Human androgen-independent prostate adenocarcinoma (PC-3); Human breast carcinoma (MCF-7); Human glioblastoma (U251)
	TsAP-1	-------	1	Human squamous carcinoma (NCIeH157); Human lung adenocarcinoma (NCIeH838)
*Buthus tumulus*	Iberiotoxin (IbTX)	Blocks K^+^ channels	1	Human glioma (U87-MG)
**Spider**				
*Lachesana tarabaevi*	Latarcin 2a	Induces pore formation and membrane destabilization	2	Human erythroleukemia (K562)
*Lycosa singoriensis*	Lycosin-1	Activates mitochondrial death pathway; Up-regulates p27	1, 2	Human fibrosarcoma (H1080); Human lung adenocarcinoma (H1299•, A549•); Human prostate carcinoma (DU145); Human colon adenocarcinoma (HCT-116); Human cervical carcinoma (HeLa•); Human hepatocellular liver carcinoma (HepG2)
*Macrothele raveni*	Whole venom	Induces DNA fragmentation; Activates caspases-3,5; Arrests cell cycle on G2/M, G0/G1; activates p21	1, 2	Human myelogenous leukemia (K562); Human breast carcinoma (MCF-7•); Human cervical carcinoma (HeLa•); Human hepatocellular carcinoma (BEL-7402)
*Phoneutria nigriventer*	Whole venom	------	1 and/or 2	Human glioma (NG97ht)

* **Cancer hallmarks:** 1. Deregulated cell proliferation, 2. Evasion of programmed cell death, 3. Sustained angiogenesis, and 4. Tissue invasion and metastasis.

• The symbol indicates the cell lines studied in xenograft tumor models.

#### 2.1.1 Scorpion venoms induce cell cycle arrest, growth inhibition, and apoptosis

The Chinese scorpion Buthus martensii Karsch (BmK; Buthidae) (since 1950, Mesobuthus martensii) was probably the first scorpion venom reported to possess antitumor properties [12]. In 1987, Zhang Futong [13] and coworkers subcutaneously administered an aliquot of full body extract of a BmK scorpion to mice bearing a reticulum cell sarcoma and mammary carcinoma (MA-737) at a dose of 0.04 g/mouse, five times per day. On the 8th day following administration, the inhibitory rate of growth was 55.5% in the reticulum cell sarcoma and 30.4% in the mammary carcinoma. It was later demonstrated that the crude venom extract from the BmK scorpion induced apoptosis in human malignant glioma (U251- MG) cells in vitro, and was especially effective at a dose of 10 mg/mL [14]. After incubation with BmK venom for 32 h and 40 h, 36.2% and 63.1% of U251-MG cells exhibited apoptosis, respectively. Also, the volume and weight of xeno-graft tumors in SCID mice were significantly reduced compared control tumor-bearing control animals after 21 d of BmK venom treatment (three times per week, 20 mg/kg intraperitoneal administration). The authors posited that ion channels are targets for BmK venom in glioma cells. Contrastingly, a study by Li et al. [15] revealed that BmK inhibited the growth (maximum effect at 24 h, 600 µg/mL) of cultured human breast cancer (MCF-7) and human hepatoma (SMMC7721) cells by inducing apoptosis (upregulating caspase-3), blocking cell cycle progression from the G0/G1 to the S phase, and downmodulating protein levels of cyclin D1 (involved in cell cycle regulation).

**Figure 4:**
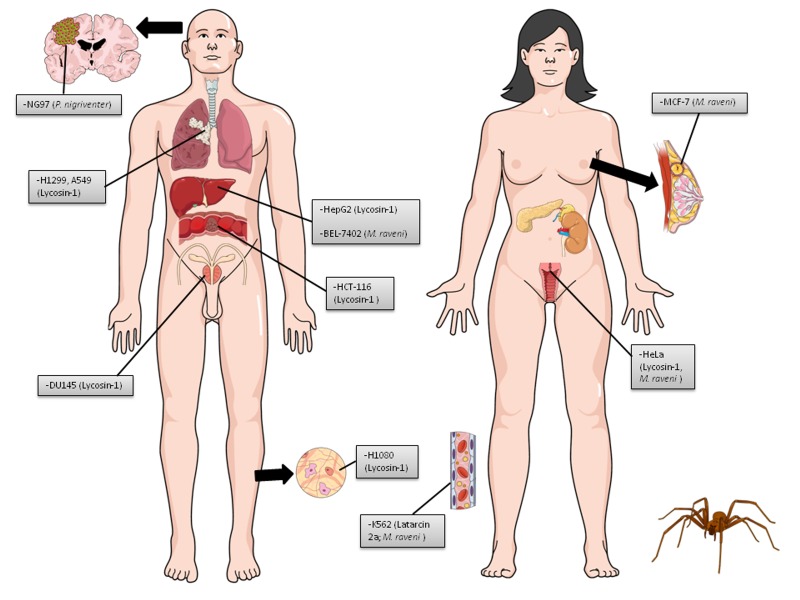
Human cancer cell lines used in spider venom and peptide studies in vitro. Images of both woman and man were inserted to represent cancers derived from the reproductive organs. There are no differences related to other lines in terms of gender. Each cell line is followed by the venom/peptide tested (in parentheses).

**Figure 5:**
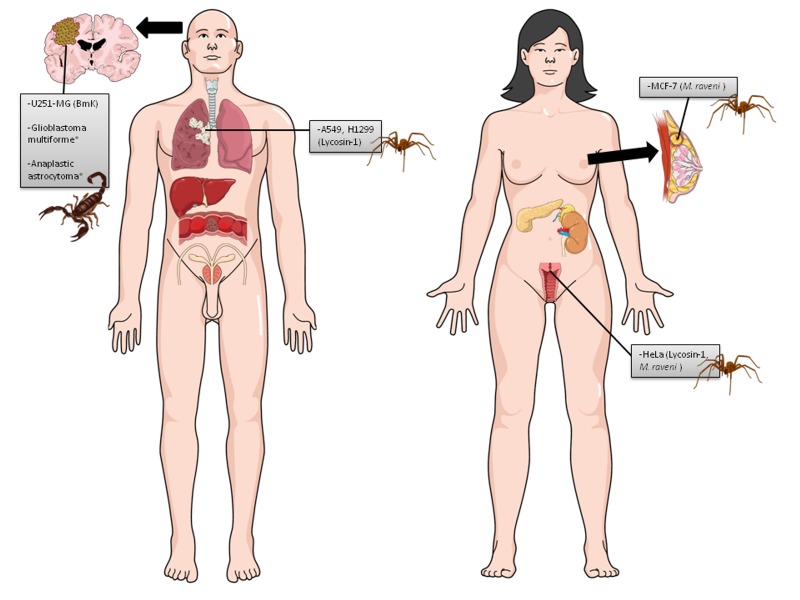
Human cancer cell lines used in scorpion and spider venom and peptide studies in vivo. Images of both woman and man were inserted to represent cancers derived from the reproductive organs. There are no differences related to other lines in terms of gender. The tumor cell lines highlighted with asterisk (*) were used in clinical trials by treating humans with toxins from scorpion. Each cell line is followed by the venom/peptide tested (in parentheses).

Alterations in cyclins, cyclin-dependent kinases (CDKs), and CDK inhibitors (CDKIs) such as p27 and p21 can lead to uncontrolled proliferation and contribute to malignant transformation (Figure 2A) [16]. The most frequent abnormalities relate to cyclin D1. Cyclin D1, CDK4, or CDK6 phosphorylates and deactivates the Rb tumor-suppressor protein (pRB) [17]. The phosphorylation of pRB results in its inactivation and the release of E2F that has been sequestered by the dephosphorylated (active) form of pRB (Figure 2A). Once liberated by pRB inactivation, E2F then proceeds to activate genes that are essential for progression into late G1 and S phase. Meanwhile, p21 and p27 inactivate the cyclin/CDK complexes, leading to the dephosphorylation of pRb and consequently to cell cycle arrest. Cyclin D1, pRb, p21, and p27 are mutated or deleted in many types of human cancer [17]. Several scorpion venoms and toxins target these cell cycle regulators and hence exhibit a capacity to curtail cancer cell proliferation.

Gao et al. [18] found that BmK venom also inhibited the growth of cultured human lymphoma (Raji and Jurkat) cells by inducing cell cycle arrest and apoptosis, while exhibiting low toxicity in human peripheral blood lymphocytes. BmK venom upregulates P27 and inactivates the PI3K/AKT (phosphatidylinositol-3 kinase/protein kinase B) signaling pathway through PTEN (phosphatase and tensin homolog deleted on chromo-some ten – a tumor-suppressor protein). The PI3K/Akt/mammalian target of rapamycin (mTOR) signaling cascade (Figure 2B) is mediated by cell surface receptors and normally stimulated by a number of growth factors, cytokines, and other extracellular stimuli [19]. It is one of the most important pathways involved in tumor growth. A common disturbance in cancer cells includes the constitutively increased activity of PI3K and a reduction in the expression or loss of PTEN (a catalytic antagonist of PI3K) [20]. The PI3K/Akt/mTOR pathway and PTEN are targets for the development of therapeutic agents for cancer treatment.

Studies have found purified peptides from BmK venom with antitumor properties. In cultured human lung cancer (A549) cells, PESV (polypeptide from BmK scorpion venom) induced cell cycle arrest in the G0/G1 phase, significantly inhibited cell proliferation, and increased the expression of PTEN [21]. In Kunming mice, high (20 mg/kg) and low (10 mg/kg) doses of PESV or PESV in combination with Rapamycin (mTOR inhibitor; 2 mg/kg) administered via gastrogavage for 14 successive days downregulated the expression of mTor and inhibited the growth of the murine hepatoma (H22) cells, leading to a reduction in tumor weight and volume [22]. PESV also inhibited cultured human leukemia (K562) cell growth and murine hepatoma (H22) tumor development in vivo (14 days treatment), decreased PI3K and AKT protein levels, and induced apoptosis [23, 24].

Evasion of apoptosis is a hallmark of most types of cancer (Figure 2) [5]. The role of several caspases and mitochondria in cell death pathways (Figure 2C), which are deregulated in cancer, is well-documented [25]. The anti-apoptotic factor Bcl-2 (B-cell lymphoma 2), an integral outer mitochondrial membrane protein, is also increased in cancer cells, while the pro-apoptotic protein BAX is downregulated [26]. Some scorpion venoms target caspases, mitochondria, Bcl-2, and BAX and may thereby contribute to cancer treatment. BmKn-2 peptide (29 µg/ml) from BmK venom killed cultured human oral squamous carcinoma (HSC-4) cells through the induction of apoptosis, as reflected by increased activated caspase-3, -7 and -9 mRNA levels [27]. BmKn-2 also induced apoptosis in HSC-4 and human mouth epidermoid carcinoma (KB) cells by activating P53 and increasing BAX/BAX and decreasing BCL-2/BCL-2 expression of both transcripts and proteins. The cells showed morphological alterations and nuclear disintegration. The peptide did not affect normal gingival (HGC) and dental pulp (DPC) cells [28]. LMWSVP peptide, from the same scorpion, dose-dependently (0.28-5.60 µg/mL; 24 h) inhibited the growth of cultured human hepatoma (SMMC7721) cells, but had no effect on the growth of cervix carcinoma (HeLa) cells. LMWSVP induced apoptosis in SMMC7721 cells by upregulating the expression of caspase-3 and downregulating the expression of BCL-2 [29].

Venom from the Egyptian scorpions Androctonus amoreuxi and Androctonus crassicauda (Buthidae) exhibited cytotoxic/antitumor properties in experimental tumor models. A. amoreuxi venom was tested in female albino mice (0.22 mg/kg, intraperitoneal administration, daily, for 14 and 30 days) in murine Ehrlich ascites and solid tumors and in cultured human breast cancer (MCF-7) cells (24, 48, and 72 h; IC50 of 0.61 µg/mL). A. crassicauda venom was tested in cultured human neuroblastoma (SH-SYSY) and MCF-7 cell lines (IC50 of 208 µg/mL and 269 µg/mL, respectively). The toxicity of these venoms in cancer cells may be related to their capability to induce necrosis or apoptosis [30, 31]. The venoms enhanced the caspase-3 expression (A. amoreuxi) or activity (A. crassicauda), while A. amoreuxi venom also induced DNA fragmentation in MCF-7 cells in vitro. Interestingly, A. amoreuxi venom ameliorated Ehrlich ascites carcinoma-induced alterations in hematological and biochemical parameters, including red and white blood cell counts [30]. A. crassicauda venom suppressed cell growth by inducing cell cycle arrest in the S-phase and cell death as a result of mitochondrial membrane depolarization [31]. A. crassicauda venom also decreased mouse brain tumor (BC3H1) cell viability by approximately 50% after exposure to 250 µg/mL of the venom for 48 h [32]. On the other hand, no significant effects of the crude venom were observed on rat fibroblast- (F2408), mouse myoblast- (CO25), transformed rat fibroblast- (5RP7), human lung carcinoma- (A549), human melanoma- (WM115), and murine fibroblast (NIH 3T3) cell lines. The same study by Caliskan et al. [32] showed that Acra3, a toxin isolated from A. crassicauda, decreased BC3H1 cell viability (IC50 of 5 µg/mL) via necrosis and apoptosis. Exposure of the cells to 0.1 and 0.5 μg/mL of Acra3 resulted in cells adopting an apoptotic morphology in a dose-dependent manner, but did not cause DNA fragmentation or increase in caspase-3 or -9 activity.

In 2007, Gupta et al. [33] reported in vitro anti-proliferative and apoptogenic activity induced by Heterometrus bengalensis Koch (Scorpionidae) (Indian black scorpion) in human leukemic (U937 – histiocytic lymphoma and K562 – chronic myelogenous leukemia) cell lines (IC50 of 41 µg/mL and 88 µg/mL, respectively; 48 h exposure). The mechanism was characterized by cell cycle arrest, membrane blebbing, chromatin condensation, and DNA degradation (typical of apoptosis). Normal human lymphocytes were not affected. The molecule of interest was subsequently purified and named Bengalin, a 72-KDa protein. Bengalin induced apoptosis in both U937 and K562 cell lines (IC50 values of 3.7 and 4.1 µg/mL, respectively), as confirmed by damaged nuclei, a sub G1 peak, and DNA fragmentation. Bengalin activates a mitochondrial death cascade, causing the loss of mitochondrial membrane potential and activating caspase-3 and -9 [34]. The toxin also decreased telomerase activity. Telomerase activity is undetectable in somatic cells, but prominent in 95% of advanced stage tumors and can contribute to the immortality of cancer cells by maintaining and stabilizing telomeres [26].

Tityus discrepans (Buthidae; Central and South America) scorpion venom and its isolated peptides neopladine 1 and neopladine 2 decrease cell viability and induce apoptosis and necrosis in human breast (SKBR3) cancer cells (5 h exposure), with a negligible effect on non-malignant monkey (MA104) kidney cells. T. discrepans venom and neopladines associate with SKBR3 cells at the cell surface, inducing FAS ligand (FASL) and BCL-2 expression and DNA fragmentation [35]. As BCL-2 suppresses apoptosis, the apoptotic effect of venom and peptides prevails over the anti-apoptotic BCL-2 effect. The anti-tumor mechanism of T. discrepans and neopladines may be via FASL. FASL expression accompanies tumor cell death; the activation of FAS signaling by the induction of FASL constitutes the trigger mechanisms of extrinsic apoptosis [36] (Figure 2C). Extrinsic apoptosis is induced by e.g., the chemotherapeutic drug methotrexate [37].

Similarly, Odontobuthus doriae (Buthidae) (yellow Iranian scorpion) venom inhibits cell growth, induces apoptosis (in-creased caspase-3 activity) and DNA fragmentation in cultured human neuroblastoma (SH-SYSY) and human breast (MCF-7) cancer cells [38, 39].

Díaz-García et al. [40] tested the effect of Rhopalurus junceus (Buthidae) (from Central America) venom against a panel of human tumor cell lines with epithelial (cervix: HeLa, SiHa, and Hep-2; lung: NCI-H292 and A549; breast: MDA-MB-231 and MDA-MB-468; colon: HT-29) and hematopoietic origin (lymphoblast: U937; myelogenous leukemia: K562; lymphoma: Raji) as well as normal cells (human fibroblast: MRC-5; canine epithelium: MDCK; monkey fibroblasts: Vero). Only the epithelial cancer cells exhibited a significant reduction in cell viability (IC50 ranging from 0.6-1 mg/mL). Among all the epithelial cancer cells, the lung (NCI-H292, A549) and breast (MDA-MB-231, MDA-MB-468) cell lines were slightly more sensitive. The scorpion venom induced chromatin condensation, increased P53 and BAX mRNA, activated caspases-3, -8, and -9, and decreased BCL-2 transcript levels. There was no effect on either normal or hematopoietic tumor cells. It is known that the tumor-suppressor protein p53 accumulates when DNA is damaged, interrupting the cell cycle at G1 for repair [41] (Figure 2A). The loss of p53 is associated with resistance of cancer cells to apoptosis (Figure 2C), contributing to the formation of tumors. The p53 tumor suppressor protein is lost due to homologous loss in 70% of colon cancers, 30-50% of breast cancers, and 50% of lung cancers [42]. Mutations in p53 or PTEN are among the most frequent causal events in many cancers, and their combined inactivation has profound consequences in terms of promoting tumor development [5]. Several scorpion venoms can beneficially modulate PTEN and/or p53 and are hence promising multi-targeting therapeutic agents.

#### 2.1.2 Inhibition of angiogenesis by scorpion venoms

Cancer cells steer the formation and growth of new blood vessels (angiogenesis) by overexpressing vascular endothelial growth factor (VEGF) and fibroblast growth factor (FGF). Increased VEGF expression is closely associated with an increase in microvessel density [43]. Inhibition of VEGF therefore is an appealing strategy for controlling angiogenesis-dependent tumor growth and metastasis.

Several studies have reported on the capability of scorpion venom peptides to suppress neovascularization and angiogenesis in tumor tissue by decreasing the level of expression of angiogenic factors. PESV (polypeptide from BmK scorpion venom) given per gavage to Kunming mice for 14 days (20 mg/kg and 10 mg/kg) induced Vegf inhibition and decreased microvessel density in murine hepatoma (H22) tumors [24]. Corroboratively, PESV reduced VEGF in cultured human lung cancer (A549) cells [21].

*A. amoreuxi* venom (0.22 mg/kg, intraperitoneal administration, daily, for 30 days) downregulated the expression of VEGF in Ehrlich solid tumors in female albino mice and decreased tumor volume and size, indicating that the venom can inhibit the neovascularization process [30].

Chlorotoxin (CTX) is a 36-amino acid peptide derived from Leiurus quinquestriatus (Buthidae) scorpion venom (Saudi Arabia), which inhibits low-conductance Cl- channels [44]. CTX and its derivatives CA4 and CTX-23 (10 μM) inhibited tube formation by human umbilical vein endothelial cells (HUVECs). CTX and CA4 also reduced tumor angiogenesis ex vivo. After incubation with the scorpion venom peptides, staining of the vascular architecture was performed in tumors that had been implanted in the brain of Wistar rats. Untreated rat glioma (F98)-implanted brain sections exhibited vessels with often irregular and hypervascularized angiogenic spots and capillaries, while CA4 or CTX (5 and 10 µM)-treated brain slices had reduced numbers of vessels that were less irregular and less dense. These data strongly suggest that CTX and CA4 are potent inhibitors of intratumoral neovascularization [45].

#### 2.1.3 Inhibition of invasion and metastasis by scorpion venoms

Tissue invasion and metastasis are hallmarks of typically advanced tumors and are associated with a negative prognosis. Both processes are characterized by loss of cell adhesion, increased motility, and proteolysis [6]. A. crassicauda venom decreased cell motility and colony formation by 60-90% in cultured human ileocecal adenocarcinoma (HCT‑8) and human colorectal carcinoma (HCT-116) cells [46]. Of note, a decrease in colony formation is an indication of inhibited proliferation in cancer cells. The same study also found that A. bicolor, A. crassicauda, and L. quinquestriatus exhibited a similar pattern of inhibition in cell motility and colony formation in human breast carcinoma (MDA-MB-231) cells.

The interaction between cells and components of the extra-cellular matrix plays a fundamental role in tumor cell invasion. Proteolysis of the extracellular matrix by matrix metalloproteinases (MMPs) facilitates this process [47]. Inhibiting the release or activity of MMPs leads to reduced motility, tumor cell invasion, and metastatic potential of malignant tumors. MMP-2 is specifically upregulated in gliomas and related cancers, but is not normally expressed in the brain. It has been demonstrated that CTX - a peptide from L. quinquestriatus scorpion venom - has an anti-invasive effect on cultured human glioma (D54-MG and CCF-STTG-1) cells, mainly due to the specific and selective interaction of this peptide with MMP-2 isoforms, but not with the MMP-1, -3, and -9 isoforms that are also expressed in glioma cells [48]. CTX exerts a dual effect on MMP-2 by inhibiting MMP-2 enzymatic activity and reducing MMP-2 surface expression. El-Ghlban et al. [49] developed a CTX-based hybrid molecule with amplified potency. It was demonstrated that the monomeric form of CTX, M-CTX-Fc (obtained by joining CTX to the amino terminus of the human IgG-Fc domain), but not CTX, decreased cell viability. M-CTX-Fc further inhibited the migration of human pancreatic cancer (PANC-1) cells and decreased MMP-2 release into the culture medium, both in a concentration-dependent manner.

Qin et al. [50] showed that CTX and CTX-modified liposomes targeted human glioblastoma (U87) and human lung (A549) carcinoma cell lines. Free CTX and CTX-modified liposomes bind to MMP-2, leading to inhibition of U87 cell migration, but not that of A549 cells. In BALB/c mice, CTX-modified liposomes (15 µg/kg, intravenous administration, five times at 3-day intervals, on days 5, 8, 11, 14, 17) also target murine metastatic breast cancer (4T1) cells, inhibiting tumor growth and deterring the incidence of lung metastases at low systemic toxicity [51]. An in vitro study by Xu et al. [45] demonstrated that CTX and its derivatives CA4 and CTX-23 peptides are highly effective in inhibiting rat glioma (F98) and human glioma (U87) cell growth, membrane extension and filopodia motility, and migration at the lowest concentration of 0.5 μM. CTX and CA4 peptides were also effective in freshly isolated primary glioma cells (30-40% reduction in cell growth). CTX and its derivatives showed no toxic effects on astrocytes and neurons. In sum, CTX, CTX-based peptide derivatives, and CTX-modified delivery systems potentially target both gliomas and non-glioma tumors that overexpress MMP-2. These inhibitory effects may prevent tumor metastasis.

Toxins from the BmK scorpion have also exhibited an effect on cell migration and metastasis. BmKCT (chlorotoxin-like peptide), cloned and sequenced from BmK by Wu et al. [52] and Zeng et al. [53], shares 68% of the amino acid sequence homology of CTX. BmKCT interacts specifically with human glioma (SHG-44) cells, but not with normal astrocytes, as a Cl‒ channel blocker [54] and inhibits the invasion and migration of rat glioma (C6) cells by antagonizing MMP-2 [55]. Similarly, the recombinant adenovirus-produced BmKCT, Ad-BmKCT, reduced rat glioma (C6) cell viability in vitro and the growth and metastasis of xenografted rat glioma (C6) tumors in female athymic nude mice following intratumoral injection of Ad- BmKCT (100 µL, 1010 viral particles, every five days) [56].

The analgesic-antitumor peptide (AGAP), a neurotoxin from BmK venom, also possesses antitumor activity. Recombinant AGAP (rAGAP) inhibited human anaplastic astrocytoma (SHG-44) and rat glioma (C6) cell proliferation, but did not result in apoptosis. The peptide led to cell cycle arrest in the G1 phase in SHG-44 cells, which was accompanied by suppression of the G1 cell cycle regulatory proteins CDK2, CDK6, and pRB as well as downmodulation pAKT and VEGF expression. rAGAP inhibited the migration of SHG-44 cells (at 10, 20 and 30 µM for 24 h) by reducing intracellular MMP-9 (but not MMP-2) [57].

#### 2.1.4 Scorpion venoms block specific transmembrane channels

There is increasing evidence that the expression of Na^+^, Ca^2+^, K^+^, Cl^‒^ [58, 59, 60] channels is altered in different cancer types and that cellular pathophysiology is influenced by the abnormal activities of these channels. Recent findings suggest that tumor cells use ion channels to support their atypical growth, cell adhesion, interaction with the extracellular matrix and invasion, by quickly adjusting cell morphology and volume (Figure 2D) [61, 14, 62, 60]. The effects of scorpion venoms have been primarily explained by the modulation of specific ion channels. Scorpion-derived peptide toxins specifically target the Na+ [63], K+ [64], and Cl‒ channels [65].

In 1983, Barhanin et al. [66] demonstrated that highly purified toxin gamma (TiTx gamma) from the venom of the Tityus serrulatus scorpion (Buthidae) (Brazilian yellow scorpion) affected Na+ channels in mouse neuroblastoma (NIE115) cells. In 1989, Kirsch et al. [67] found that TsIV-5 toxin (500 nM), also isolated from T. serrulatus venom, and blocked the whole-cell and single-channel Na+ current in mouse neuroblastoma (N18) cells. More recently, Guo et al. [68] demonstrated that TsAP-2, a peptide whose structure was deduced from cDNAs cloned from a venom-derived cDNA library of T. serrulatus, inhibited the growth of five human cancer cell lines: squamous cell carcinoma (NCIeH157), lung adenocarcinoma (NCIeH838), androgen-independent prostate adenocarcinoma (PC-3), breast carcinoma (MCF-7), and glioblastoma (U251). The synthesized TsAP-1 peptide, also deduced from the T. serrulatus cDNA library, was active in only two of the five human cancer cell lines (NCIeH157 and NCIeH838). In the same study, the analogues of each peptide known as TsAP-S1 and TsAP-S2, were also successfully synthesized. These analogues were specifically designed to enhance the cationicity of each natural peptide. Cationic linear peptides are known for their anticancer properties [69]. The potency of TsAP-1 in NCIeH157 and NCIeH838 cancer cells was enhanced more than 30-fold when their cationicity was increased (i.e., TsAP-S1), and the potency of TsAP-2 in all five cancer cell lines was enhanced by 3.5-8.5-fold compared to the native peptide. These results illustrate that drug candidates obtained from scorpion venom can be optimized to yield greater pharmacodynamic efficacy.

There is an upregulation of Cl^‒^, K^+^, and Na^+^ channels in glioma cells [58, 59]. Excessive activity of a Cl^‒^ ion channel, which is absent in normal brain tissue, has been described in malignant gliomas [65]. This glioma-specific Cl‒ channel can shape glioma cell morphology, foster proliferation and migration, and regulate apoptosis [70, 71]. It has been demonstrated that CTX-modified liposomes targeted human glioblastoma (U87) cells, activating the receptor-chloride channel associated protein ClC-3 via binding to MMP-2, leading to the inhibition of cell migration and Cl‒ currents [50].

An iodine 131 (I131) radioconjugate of the synthetic CTX (TM-601), I131-TM-601, has potential antiangiogenic and antineoplastic activities. Since CTX specifically binds to tumor cells overexpressing MMP-2, the I131-TM-601 may be used as a radioimaging agent [72] while concurrently relaying a tumor-specific, cumulative radiocytotoxic dose of I131. In addition, TM-601 alone, similar to native CTX, could inhibit or kill the tumor cells and reduce angiogenesis due to its ability to bind to and inhibit MMP-2, contributing to the antineoplasic effect of I131-TM-601 [73]. Phase I human trials [74] evaluated the safety, biodistribution, and dosimetry of intracavitary-administered 131I-TM-601 (synthetic CTX) [55] in patients with recurrent glioma (17 with glioblastoma multiforme and one with anaplastic astrocytoma). A single dose of 10 mCi 131I-TM-601 (0.25-1.0 mg TM-601) was tolerated and exerted an antitumor effect. 131I-TM-601 bound the tumor periphery and demonstrated long-term retention in the tumor, with minimal uptake in other organ systems. On day 180, four patients had a radiographically stable disease, and one patient experienced a partial response. Two of these patients improved further and did not display any evidence of disease for more than 30 months. A phase II trial with this agent using higher doses of radioactivity and repeated local administration is underway [75], available in https://clinic-altrials.gov/ct2/show/NCT00683761 and http://adisinsight.springer.com/trials/700034613).

In a study by Fan et al. [76], the mature peptide coding region of BmKCT (from the venom of the BmK scorpion; which interacts specifically with glioma cells as a Cl‒ channel blocker) was amplified by PCR from the full-length cDNA sequence of BmKCT (screened from the venomous gland cDNA library of BmK scorpion). In the same study, the recombinant GST- CTX protein was also cloned. Both GST-BmKCT as well as GST-CTX selectively targeted to tumor tissue following injection of the fluorescent Cy5.5 or radioactive 131I conjugates into rats. After 18 days of intraperitoneal administration of both the recombinant proteins in tumor-bearing female, Sprague Dawley rats, rat glioma (C6) tumor growth and metastasis were inhibited.

A previous study found a correlation between the activity of K+ channels and the proliferation of glioma cells and xeno-grafted tumors [77]. A variety of K+ channel blockers, including iberiotoxin (IbTX; a specific KCa channel blocker), purified from the Eastern Indian red scorpion Buthus tamulus (Buthidae), significantly inhibited the proliferation of cultured human glioma (U87-MG) cells [78]. However, Kv and KATP channel blockers induced more significant effects than IbTX, indicating that these channels play a more important role than KCa channels in the proliferation of U87-MG cells. BmKKx2, a 36-residue toxin from the BmK scorpion, is a potent human Ether-à-go-go-Related Gene (hERG) K+ channel blocker. BmKKx2 can reduce the proliferation of human myelogenous leukemia (K562) cells and cause cell cycle arrest in the G1 phase, demonstrating its potential use in treating leukemia [79]. BmKKx2 (200 nM for 48 h) suppressed proliferation, enhanced erythroid differentiation as well as differentiation-dependent apoptosis in cultured K562 cells. Previous studies showed that the leukemia cells tended to be more sensitive to apoptosis inducers during the differentiation process [80]. BmKKx2 had no effect on the erythroid differentiation of K562 cells after hERG channel knockdown, confirming that BmKKx2 was able to accelerate K562 cell differentiation through interacting with hERG channels.

It is clear that scorpion venoms possess a selective and differentiated toxicity against cancer cells by acting on multiple targets. The mechanisms, while diverse, affect growth/survival pathways, cell death pathways, angiogenesis, migration/metastasis, and/or ion channels.

### 2.2 Spider venoms

Literature about the effects of spider venoms on cancer cells is not as broad as that of scorpion venoms, and there is sparse scientific evidence for their potential in cancer therapy. Spiders are the most diverse group of arthropods (38,000 described species). Nevertheless, relatively few toxins have been studied so far [1], making this an opportunistic field for exploration [12]. The major components of most spider venoms are small, stable disulfide-bridge peptides that are resistant to proteolytic degradation. In addition, many of these peptides have high specificity and affinity towards molecular targets that are of therapeutic importance. The combination of bioactivity and stability has rendered spider venom peptides valuable as pharmacological tools and as (potential) leads for drug development [81].

Peptides are considered a novel class of anticancer agents with the capability to specifically target cancer cells while exhibiting lower toxicity in normal tissues [82]. Spider peptides have demonstrated general cytotoxicity, including antifungal, antimicrobial, hemolytic, and anticancer activity in several cell lines and tumor models (Figures 4 and 5). Latarcins, linear cytolytic peptides from Lachesana tarabaevi (Mierenjagers, Zodariidae) Central Asian spider venom, show anticancer potential [83]. Latarcin 2a (Ltc2a; GLFGKLIKKFGRKAISYAVKKARGKH-COOH), a short linear antimicrobial and cytolytic peptide, induced the formation of large pores in bilayers [69]. Vorontsova et al. [84] demonstrated that Ltc2a possesses in vitro cytotoxicity against human erythroleukemia (K562) cells. Interestingly, apoptosis was not activated by the peptide. Penetration of Ltc2a to the cytoplasmic leaflet of the plasma membrane and formation of membrane pores involving several peptides per pore are the most evident mechanism, but the whole sequence of events occurring at the membrane still needs to be clarified. Ltc2a was cytotoxic for erythrocytes (EC50 = 3.4 μM), leukocytes (EC50 = 19.5 μM), and K562 cells (EC50 = 3.3 μM). The peptide induced membrane blebbing and swelling of K562 cells, followed by cell death.

The peptide Lycosin-1, isolated from the venom of Lycosa singoriensis (Lycosidae; from Central and Eastern Europe), exhibits a linear amphipathic alpha-helical conformation and inhibits tumor cell growth in vitro and in vivo [85]. Lycosin-1 (40 µM) caused more than 90% cell death in the following human cancer cell lines: fibrosarcoma (H1080), lung adenocarcinoma (H1299, A549), prostate carcinoma (DU145), colon adenocarcinoma (HCT-116), cervix carcinoma (HeLa), and hepatocellular carcinoma (HepG2). In contrast, treatment of non-cancerous human liver (L02) cells, non-transformed mouse skin epidermal (JB6) cells, and erythrocytes with lycosin-1 caused less than 25% cell death. The peptide moved across the plasma membrane, being internalized, and activated intrinsic apoptosis (i.e., mitochondrial pathway). Also, lycosin-1 upregulated P27 and inhibited cell proliferation.

In vivo investigations have been performed in human A549, H1299, and HeLa xenograft-bearing nude mice. Lycosin-1 (50, 100, and 200 µg per mouse, daily, for 18 days) inhibited growth of the implanted tumors in a dose-dependent manner, with little apparent systemic toxicity. In addition, the cells in lycosin-1-treated tumor tissues displayed clearly chromosomal condensation and nuclear shrinkage, a typical morphological feature associated with apoptosis. Apoptosis was further confirmed by TUNEL staining [85].

The venom of the Macrothele raveni spider (Hexathelidae; from Asia) potently suppressed cell growth in human myelogenous leukemia (K562) cells and had a low inhibitory effect on human lymphocytes, suggesting that the venom is relatively selective for leukemia cells. The venom had a dose-dependent inhibitory effect with an IC50 of 5.1 µg/mL. Venom-treated K562 cells showed morphology indicators that were consistent with apoptosis, including condensation of nuclei, DNA fragmentation, and caspase-3 and -8 activation [86]. The venom of M. raveni also exhibited dose-dependent antitumor activity (10, 20, and 40 µg/mL, 24 h incubation) in human breast carcinoma (MCF-7) cells, affecting cell viability, inhibiting DNA synthesis, and inducing apoptosis and necrosis. MCF-7 cells treated with the venom were arrested in the G2/M and G0/G1 phase. In addition, the spider venom activated the expression of P21 [87]. In cultured human hepatocellular carcinoma (BEL-7402) cells, M. raveni venom inhibited proliferation and DNA synthesis and induced apoptosis and cell cycle arrest in the G0/G1 phase [88].

In terms of in vivo studies, the size of human breast carcinoma (MCF-7) tumors in nude mice was reduced after 21 days of treatment with M. raveni venom (1.6, 1.8, and 2 µg/g; daily tail vein injection) [87]. Moreover, marked morphological changes, inhibition of proliferation, and caspase-3 upregulation were observed in human cervix carcinoma (HeLa)- bearing nude mice treated with M. raveni venom. Tumor size decreased after 3 weeks of treatment with venom (1.0, 2.0, and 4.0 µg/g, tail vein injection) [89].

Phoneutria nigriventer spider (Ctenidae; from tropical South America) venom (PNV) contains peptides that affect the Ca2+, K+, and Na2+ ion channels [90]. Furthermore, the Phα1β peptide from PNV has an analgesic effect in a cancer pain model [91]. However, to our knowledge, the effects of PNV in tumor cells have not yet been elucidated. Nevertheless, the venom constitutes an interesting source of potential drug candidates for the treatment of glioma owing to its ion channel blocking properties.

**Figure 6:**
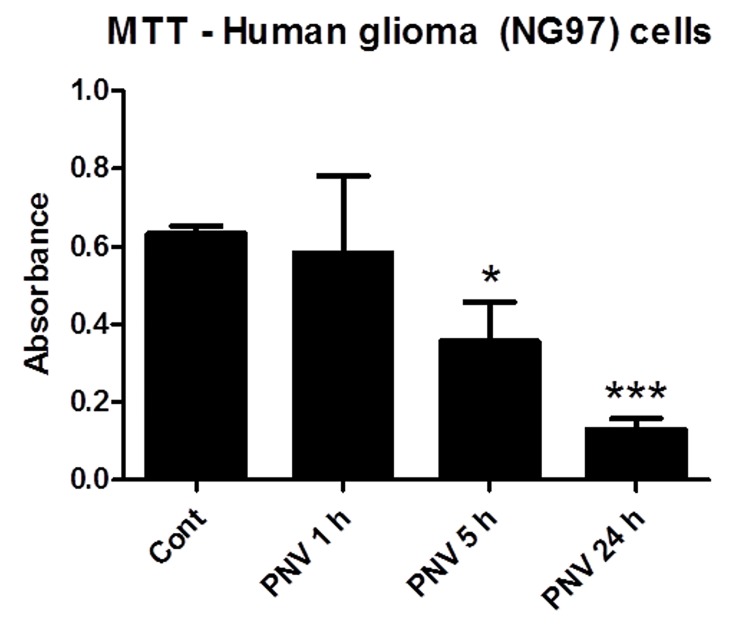
Viability (MTT) assay with cultured human glioma (NG97) cells following exposure to Phoneutria nigriventer venom (PNV; 14 µg/mL) for 1, 5, and 24 h (controls remained in the IMDM medium). * *p* < 0.05, *** *p* < 0.001 compared to control cells (ANOVA followed by Dunnett’s multiple comparison post-test; three sets of experiments were used for comparison; *p* of ≤ 0.05 was considered significant).

PNV changes blood-brain barrier (BBB) permeabilization [92, 93, 94, 95, 96] and selectively affects astrocytes. It has been demonstrated that PNV induces edema in astrocyte end-feet [92, 93] and increases glial fibrillary acidic protein (Gfap), S100 [97], aquaporin-4 [98] and connexin 43 (Cx43) [95, 99] in rat astrocytes in vivo and/or in vitro. All of these proteins are important astrocytes markers. In culture, PNV induced a Ca2+-mediated response; changed stress fibers and F/G-actin balance; and induced profound alterations in astrocyte morphology [99]. In addition, the venom increased Na+/K+-ATPase [99] and Pten expression [94] and reduced PI3K and Akt levels (unpublished results). Aberrant expression and the altered activity of Na+/K+-ATPase subunits have been implicated in the development and progression of many cancers [100]. Taken together, these data suggest that the venom contains peptides that can target glioma cells, which are developed from glia cells, and especially transformed astrocytes [101]. In fact, preliminary data from our research group demonstrated that PNV decreased human glioma (NG97) cell viability after 5 and 24 h of venom exposure (Figure 6). It is possible that PNV also inhibits glioma cell migration and metastasis, since the venom impairs the cytoskeleton of astrocytes and cell morphology. Experiments to elucidate the anticancer mechanism of PNV and to isolate the molecule(s) responsible for these effects are in progress.

Taken altogether, it has been shown that scorpion and spider venoms and purified peptides are highly specific for multiple targets (Table 1) involved in several key hallmarks of cancer (Figure 2). Anticancer drugs generally affect only one aspect of cancer cell biology, namely cell division. Scorpion and spider venom constituents affect not only cell growth and division, but also other important components of tumor cell behavior/tumor development, including angiogenesis, cell morphology, motility and migration. The venom constituents further target numerous specific proteins and pathways important in tumor cell metabolism and homeostasis. A clinically relevant point is that several scorpion and spider toxins have no cytotoxic effects on normal cells, including white blood cells, which is a common side effect of several forms of chemotherapy [18, 28, 30, 32, 33, 35, 40, 45, 54, 85]. However, few drug candidates from venoms have been used in the clinical setting to date, making this a challenge in translational research.

## 3 Animal venoms and translational research: a challenge

**Figure 7:**
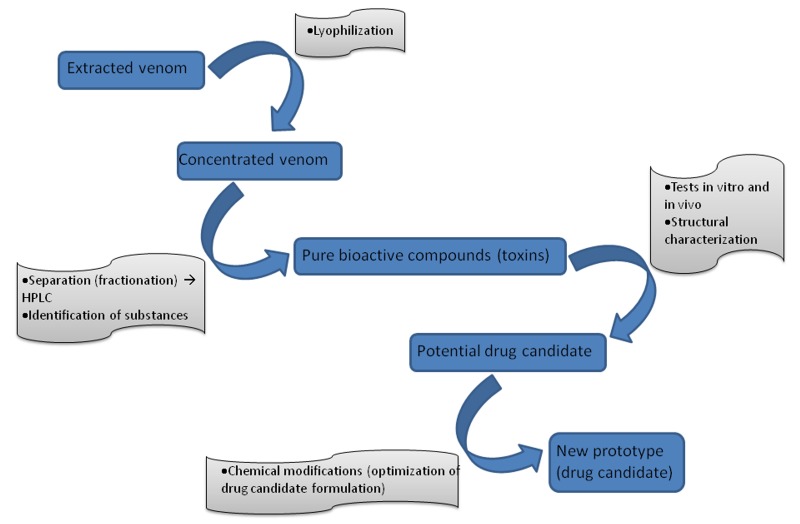
Generic process of discovering new drugs through the screening of natural products with biological activity. HPLC - high performance liquid chromatography.

Currently, more than 50% of the drugs used worldwide, including chemotherapeutic drugs, are derived from natural products [102]. There are many examples of compounds from venomous animals, such as snakes, spiders, scorpions, caterpillars, bees, insects, wasps, centipedes, ants, toads, and frogs, demonstrating potential biotechnological or pharmacological application [12, 103]. Whereas molecules derived from bacteria, fungi, marine organisms, and plants are often used in clinical practice, molecules derived from animals (mainly arthropods) are rarely used as drug prototypes or in clinical trials and practice. This may be because molecules from animals are difficult to produce commercially (Figure 7), as they are large and complex (frequently peptides or proteins) and difficult to synthesize and modify by synthetic chemistry [26]. This renders the optimization of drug candidates and commercial production very tedious and expensive.

The pharmaceutical industry has been responsible for the most important therapeutic advances of the last 50 years [26]. The entire process of bringing a new medicine to market entails discovery, preclinical research (in vitro and in vivo), clinical trials, approval by regulatory agencies, and launch [26]. This is an expensive and time-consuming process which can take around 10-15 years. The pharmaceutical companies represent a highly monopolized and profitable sector of the economy that requires major investment in research and development. At the same time, by the logic of business, the industry is interested in reducing costs and producing more profitable drugs [104]. It is possible that the difficulties and high costs involved in obtaining pure bioactive prototypes from arthropods have discouraged the pharmaceutical industry in pursuing these leads that in turn contributed to the limited clinical use of these compounds.

Furthermore, the market share for the pharmaceutical industry in developing countries is extremely small: only 7.7% for Africa, Asia, and Australia combined and 3.8% for Latin America [104]. Many countries with a rich stock of venomous animals are located in those regions, where universities and research institutes conduct research studies on the venoms. Collaboration with the pharmaceutical industry is not common, however, compared with the established, close relationships between universities and companies seen in developed countries [105]. A study by Caramori and Berraviera [104] recently described this issue as follows:

“The broad biodiversity of venomous animals in Brazil is widely known, but the public research community dedicated to drug discovery and development, namely universities and research centers, has so far been confined to experimental laboratories, working in an isolated and fragmented fashion. As a result, basic research findings are published but rarely move forward.”

To improve this situation, firstly the demand from companies in developing countries should be stimulated and, secondly, the provision of knowledge by the universities and institutes should be increased [105]. Inadequate collaboration between universities or research centers and interested companies in these countries can explain, at least in part, the difficulty of advancing the venoms to clinical trials.

In addition, government actions and programs are needed to promote translational research and guide university-based biomedical research in developing countries. Efforts to channel funds for biomedical research are fundamental to the development of translational research. Creating centers and institutes specifically aimed at the expansion of translational research in developing countries are also of great importance. These centers can connect basic research, technological development, clinical research, and product commercialization and regulation. Barraviera [106] has suggested the creation of a Center for Bioprospecting and Clinical Trials in Brazil as a way of overcoming the gap between basic and clinical research. According to the author, such a center would be dedicated to prospecting bioactive molecules, conducting preclinical and clinical trials, transferring technology to both public and private bodies, and accelerating the production of previously identified drug candidates that are currently at more advanced developmental stages, such as many toxins from scorpion and spiders, for purposes of investigating lead compounds and treating cancer. Considering the rich variety of venomous animals found in Brazil, the creation of a center with these objectives is most encouraging. The involvement of developing countries in the translational research environment is of utmost importance.

In summary, in spite of many promising initial and pre-clinical studies, the clinical application of scorpion and spider toxins for the treatment of cancer remains a challenge, yet needs to move forward. The formation and strengthening of public-private and public-public partnerships, the application of public funds, the creation of centers for translational research expansion, the development of local businesses, and specifically the encouragement of partnerships between universities and the pharmaceutical industry are imperative to advance the translational research movement in developing countries where these venoms are sourced and studied.

## ABBREVIATIONS

AIF: Apoptosis-inducing factor;
Akt: Protein kinase B;
BBB: Blood-brain barrier;
Bcl-2: B-cell lymphoma 2;
BmK: Buthus martensii Karsch;
BmKCT: Chlorotoxin-like peptide;
CDKIs: CDK inhibitors;
CDKs: cyclin-dependent kinases;
ClC-3: Receptor-chloride channel associated protein;
CTX: Chlorotoxin;
Cx43: Connexin 43;
Cyt-c: Cytochrome-c;
FADD: Associated protein with death domain;
FDA-US: Food and Drug Administration;
FGF: fibroblast growth factor;
GFAP: Glial fibrilar acidic protein;
GPCR-G: protein-coupled receptors;
HPLC: High Performance Liquid Chromatography;
IbTX: Iberiotoxin;
Ltc2a: Latarcin 2a;
MMPs: Matrix metalloproteinases;
mTOR: Mammalian target of rapamycin;
PESV: Polypeptide from BmK scorpion venom;
PESV: Polypeptide from BmK scorpion venom;
PI3K: Phosphatidylinositol-3 kinase;
PIP2: Phosphatidylinositol 4,5-bisphosphate;
PIP3: Phosphatidylinositol 3,4,5-trisphosphate;
PNV: Phoneutria nigriventer spider venom;
pRB: Rb tumor-suppressor protein;
PTEN: Phosphatase and tensin homolog deleted on chromosome ten;
RTK: Receptor tyrosine kinases;
TR: Translational research;
VEGF: Vascular endothelial growth factor.
